# Fibroblast growth factor 21 deficiency exacerbates chronic alcohol-induced hepatic steatosis and injury

**DOI:** 10.1038/srep31026

**Published:** 2016-08-08

**Authors:** Yanlong Liu, Cuiqing Zhao, Jian Xiao, Liming Liu, Min Zhang, Cuiling Wang, Guicheng Wu, Ming-Hua Zheng, Lan-Man Xu, Yong-Ping Chen, Moosa Mohammadi, Shao-Yu Chen, Matthew Cave, Craig McClain, Xiaokun Li, Wenke Feng

**Affiliations:** 1 School of Pharmacy and First Affiliate Hospital, Wenzhou Medical University, Wenzhou, Zhejiang, 325027, China; 2 Alcohol Research Center, University of Louisville School of Medicine, Louisville, KY, 40202, USA; 3 Institute of Life Sciences, Wenzhou University, Wenzhou, Zhejiang, 325035, China; 4 School of Life Sciences, Northwest University, Xi’an, Shaanxi, 710069, China; 5 Three George Central Hospital, Chongqing, 404000, China; 6 Department of Biochemistry and Molecular Pharmacology, New York University School of Medicine, New York, NY, 10016, USA; 7 Robley Rex VA Medical Center, Louisville, KY, 40206, USA

## Abstract

Fibroblast growth factor 21 (FGF21) is a hepatokine that regulates glucose and lipid metabolism in the liver. We sought to determine the role of FGF21 in hepatic steatosis in mice exposed to chronic alcohol treatment and to discern underlying mechanisms. Male FGF21 knockout (FGF21 KO) and control (WT) mice were divided into groups that were fed either the Lieber DeCarli diet containing 5% alcohol or an isocaloric (control) diet for 4 weeks. One group of WT mice exposed to alcohol received recombinant human FGF21 (rhFGF21) in the last 5 days. Liver steatosis and inflammation were assessed. Primary mouse hepatocytes and AML-12 cells were incubated with metformin or rhFGF21. Hepatic genes and the products involved in *in situ* lipogenesis and fatty acid β-oxidation were analyzed. Alcohol exposure increased circulating levels and hepatic expression of FGF21. FGF21 depletion exacerbated alcohol-induced hepatic steatosis and liver injury, which was associated with increased activation of genes involved in lipogenesis mediated by SREBP1c and decreased expression of genes involved in fatty acid β-oxidation mediated by PGC1α. rhFGF21 administration reduced alcohol-induced hepatic steatosis and inflammation in WT mice. These results reveal that alcohol-induced FGF21 expression is a hepatic adaptive response to lipid dysregulation. Targeting FGF21 signaling could be a novel treatment approach for alcoholic steatohepatitis.

Alcoholic fatty liver disease (AFLD) is characterized by excessive fat accumulation in the liver, and it may progress to more harmful stages of liver injury, including steatohepatitis, fibrosis, cirrhosis, and even malignancy. Hepatic steatosis has also been observed in patients with obesity and diabetes and in experimental animals fed with a high fat diet. The control of hepatic lipid metabolism is a complex process. Fatty acid synthesis, uptake, oxidation and release are basic regulatory mechanisms responsible for fat accumulation in the liver. Extensive studies have identified key regulators in these processes, including hormones such as insulin and glucagon, transcription factors and regulatory molecules such as peroxisome proliferator-activated receptor α (PPARα)^1^, sterol regulatory element-binding protein 1c (SREBP1c)^2^, and sirtuin 1(Sirt1)^3^. Chronic alcohol ingestion is believed to cause enhanced hepatic lipogenesis and impaired fatty acid β-oxidation by dysregulation of key hepatic factors such as PPARα, SREBP1c, PPARγ coactivator α (PGC1α), Sirt1 and AMP-activated kinase (AMPK)^4^. During alcohol exposure, production of reactive oxygen species is enhanced due to the up-regulation of cytochrome P450 2E1 (Cyp2e1)^5^. Although profound changes in pancreas-produced insulin and glucagon (known as causative extrahepatic hormones) have been described, little is known as to whether the paracrine and endocrine signals for metabolic regulation of the liver itself participate in these alcohol-induced alterations in lipid metabolism.

Fibroblast growth factor (FGF21) is a potential metabolic regulator^6^. Unlike typical FGFs, FGF21 lacks heparin binding, and therefore serves as an endocrine factor. FGF21 is activated by binding to FGF receptors and a unique co-receptor, β-Klotho, which is expressed abundantly only in certain metabolic tissues, such as liver, adipose tissue and pancreas^7^. The mechanisms underlying the action of FGF21 in the regulation of hepatic lipid homeostasis have been explored in animals. FGF21 deficient mice display an abnormal lipid response, including attenuated triglyceride clearance and enhanced lipogenesis in the liver during methionine-choline deficient (MCD) diet and high fat diet feeding in mice^8^. Alcoholic liver disease (ALD), in particular in its early stages including AFLD and alcoholic steatohepatitis (ASH), is characterized by hepatic intracellular lipid accumulation. These similarities suggest that FGF21 may be involved in the alcohol-induced hepatic fat accumulation and liver injury. A recent study showing that FGF21 plays a role in alcohol preference in mice further indicates the importance of FGF21 in alcohol related disorders^9^. In the present study, we investigated the role of FGF21 signaling in hepatic lipid regulation and inflammation in ALD. By using global FGF21 knockout (KO), we demonstrate that FGF21 signaling is involved in the development/progression of experimental alcoholic liver disease.

## Results

### Mice with alcohol-induced steatosis and liver injury have increased serum FGF21

**Figure 1:**
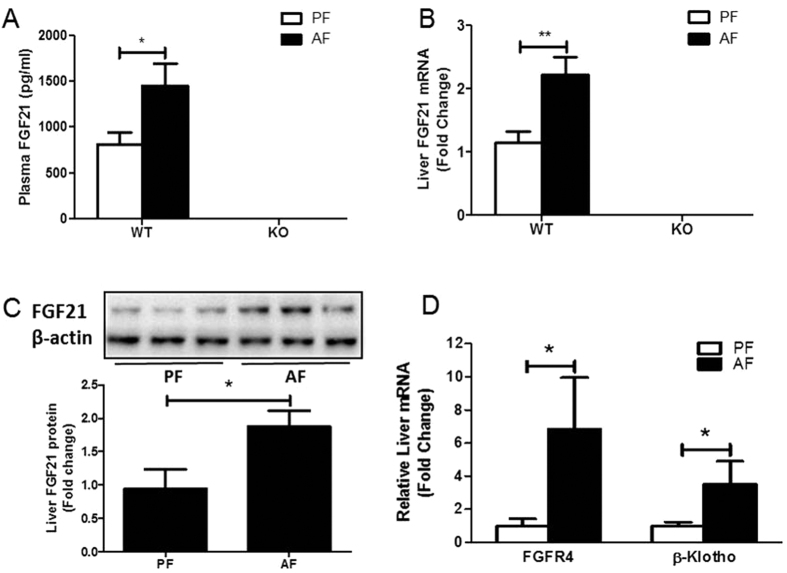
Alcohol exposure increases FGF21 expression. (**A–D**): Wild type (WT) and FGF21 KO (KO) mice were treated as described in Material and Methods. (A) Plasma FGF21 concentration. (**B**) Relative liver mRNA levels of FGF21. (**C**) Hepatic FGF21 protein levels. (**D**) Relative liver mRNA levels of β-Klotho and FGFR4.

Exposure of 8- to 10-week-old C57BL/6J mice to the Lieber DeCarli liquid diet containing 5% alcohol for 4 weeks resulted in a significant increase in FGF21 expression in the serum and liver (Fig. 1A–C). FGF21 knockout mice served as controls (Fig. 1A,B). FGFR4 and β-Klotho were also markedly elevated in the liver in alcohol-fed mice (Fig. 1D). Alcohol markedly increased FGF21 gene expression in primary hepatocytes isolated from the mice exposed to alcohol (Supplementary Figure 1A). In contrast, epididymal white adipose tissue isolated from alcohol-exposed mice did not show an alteration in FGF21 gene expression compared to controls (Supplementary Figure 1B). These results suggest that alcohol exposure activates FGF21 signaling in hepatocytes.

### Serum FGF21 levels are increased in alcoholic steatohepatitis (ASH) patients

To determine whether humans with ALD have elevated FGF21 levels similar to mice with alcohol-induced hepatic steatosis and liver injury, we measured serum FGF21 concentrations in 24 patients with ASH, in 20 patients with alcoholic cirrhosis (AC) with no fatty liver on ultrasound/CT scan, and in 26 nondrinking healthy subjects with no liver disease. Baseline clinical, biochemical and demographic information is shown in Supplementary Table 1. As shown in Supplementary Figure 1C, patients with ASH had FGF21 values that were six times greater than those seen in healthy controls, while stable cirrhotics had values that were not significantly different from controls.

### Alcohol feeding increases liver steatosis and injury

**Figure 2:**
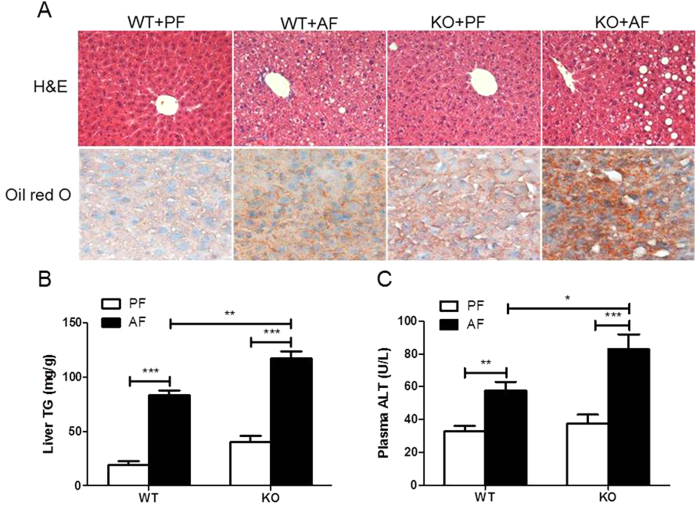
FGF21 ablation exacerbates chronic alcohol-induced liver steatosis and injury. Mice were treated as described in Material and Methods. (**A**) Hematoxylin and eosin (H&E, upper panel), and Oil red O (lower panel) staining of liver sections. (**B**) Liver TG concentrations. (**C**) Plasma ALT levels.

The alcohol-fed mice developed fatty liver, as demonstrated by histological analyses of liver sections (Fig. 2A) and hepatic triglyceride (TG) levels (Fig. 2B). Alcohol exposure caused hepatocellular damage, as indicated by elevated plasma alanine aminotransferase (ALT, Fig. 2C) and hepatic apoptosis as determined by TUNEL (terminal deoxynucleotidyl transferase dUTP nick end labeling) staining compared to PF controls (Supplementary Figure 2).

### FGF21 ablation exacerbates chronic alcohol-induced liver steatosis and injury

Next, we tested whether FGF21 plays a role in the development of ALD. As shown in Supplementary Table 2, alcohol exposure significantly increased liver weight and liver/body weight ratio. These increases were more pronounced in FGF21 KO mice, indicating a likely fat accumulation increase in FGF21 KO mice. Alcohol exposure increased the levels of plasma free fatty acid (FFA) and TG in WT mice. Plasma TG levels were further increased by alcohol exposure in FGF21 KO mice. There were no changes in plasma levels of cholesterol and insulin between WT and FGF21 KO mice. Food intake and blood alcohol concentrations were not different between WT and FGF21 KO mice (data not shown).

However, FGF21 KO mice exhibited a more severe hepatic steatosis (Fig. 2A). Confirming these findings, hepatic TG was markedly increased in FGF21 KO mice (Fig. 2B). Plasma ALT levels were further increased in FGF21 KO mice (Fig. 2C). These findings strongly suggest that FGF21 may be playing a mechanistic role in the hepatic defense to alcohol-induced steatosis and hepatotoxicity.

### FGF21 ablation increases hepatic lipogenesis by alcohol exposure

The control of hepatic lipid metabolism is an intricate process. In both WT and KO mice, chronic alcohol feeding significantly increased the gene expression of cluster of differentiation 36 (CD36), a fatty acid transporter in the liver, but not fatty acid transport protein FATP2 and FATP5 (Supplementary Figure 3A). There was no change in hepatic expression of the genes responsible for very low-density lipoprotein (VLDL) assembly (Supplementary Figure 3B).

**Figure 3:**
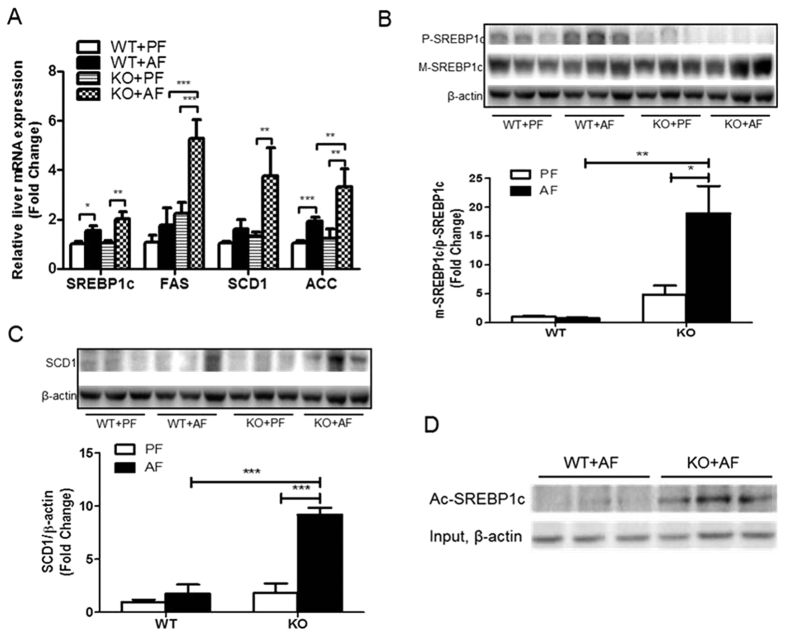
FGF21 ablation increases hepatic lipogenesis by alcohol exposure. Mice were treated as described in Material and Methods. (**A**) Relative liver mRNA levels of SREBP1c, FAS, SCD1 and ACC. (**B**) Hepatic protein levels of precursor (p-SREBP1c) and mature (m-SREBP1c) forms of SREBP1c (upper panel), and the ratio of m-SREBP1c/p-SREBP1c (lower panel). (**C**) Hepatic SCD1 protein levels. (**D**) Acetylated SREBP1c levels.

In agreement with previous studies^2^, alcohol exposure increased hepatic gene expression of SREBP1c, a transcription factor which plays a critical role in the control of lipogenic gene expression (Fig. 3A). Accordingly, the gene expression of well-known targets of SREBP1c, fatty acid synthase (FAS), stearoyl-CoA desaturase-1 (SCD1) and acetyl-CoA carboxylase (ACC), was increased by alcohol exposure in the livers of WT mice; and these increases were more pronounced in the KO mice (Fig. 3A). The precursor level of SREBP1c was significantly increased in AF WT mice, but decreased in AF KO mice (Fig. 3B). However, the mature form of SREBP1c tended to be increased in the KO mice resulting in a drastically increased ratio of mature to precursor SREBP1c in AF KO mice, indicating a significant activation of SREBP1c. The levels of the SCD1 protein were markedly increased in the KO mice by AF (Fig. 3C). Furthermore, in the KO mice, alcohol increased SREBP1c protein acetylation level (Fig. 3D), which increases SREBP1c protein stability and activity^10^.

**Figure 4:**
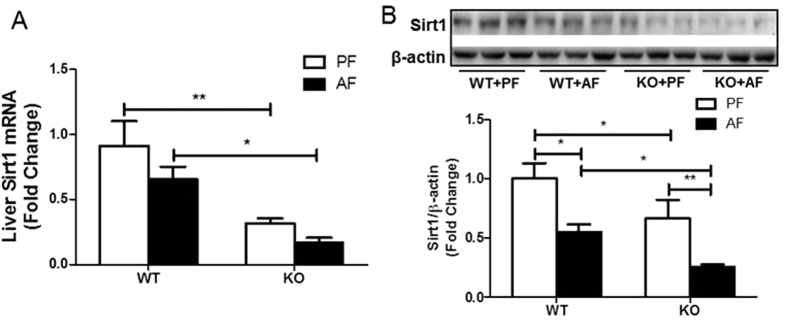
FGF21 deficiency decreases alcohol-mediated Sirt1 activity. Liver Sirt1 mRNA (**A**) and protein (**B**) in the liver of mice treated as described in Material and Methods.

Previous studies indicated that Sirt1 is an oxidized nicotinamide adenine dinucleotide (NAD+)-dependent deacetylase, which tightly regulates fatty acid metabolism through multiple nutrient sensors including SREBP1c and PGC-1α^10^,^11^. Sirt1 activation was impaired more severely in the KO mice. Hepatic Sirt1 transcript expression was significantly decreased in the PF KO mice, and was further decreased in the AF KO mice (Fig. 4A). The reduction was also observed at protein levels (Fig. 4B). We next sought to determine whether FGF21 expression is required in Sirt1 expression. It has been shown that metformin increases Sirt1 expression in hepatocytes^12^. Incubation with metformin for 8 hours resulted in a significant increase in Sirt1 protein expression in primary hepatocytes isolated from WT mice, but the increase was not as great in the KO mice (Supplementary Figure 4A), indicating a requirement for FGF21 in the metformin induction of Sirt1. Furthermore, rhFGF21 treatment markedly induced Sirt1 nuclear translocation in AML-12 cells (Supplementary Figure 4B). These results suggest a possible role for FGF21 in the Sirt1-mediated regulation of hepatic lipogenesis induced by chronic alcohol exposure.

### FGF21 knockout inhibits alcohol-regulated hepatic fatty acid β-oxidation

**Figure 5:**
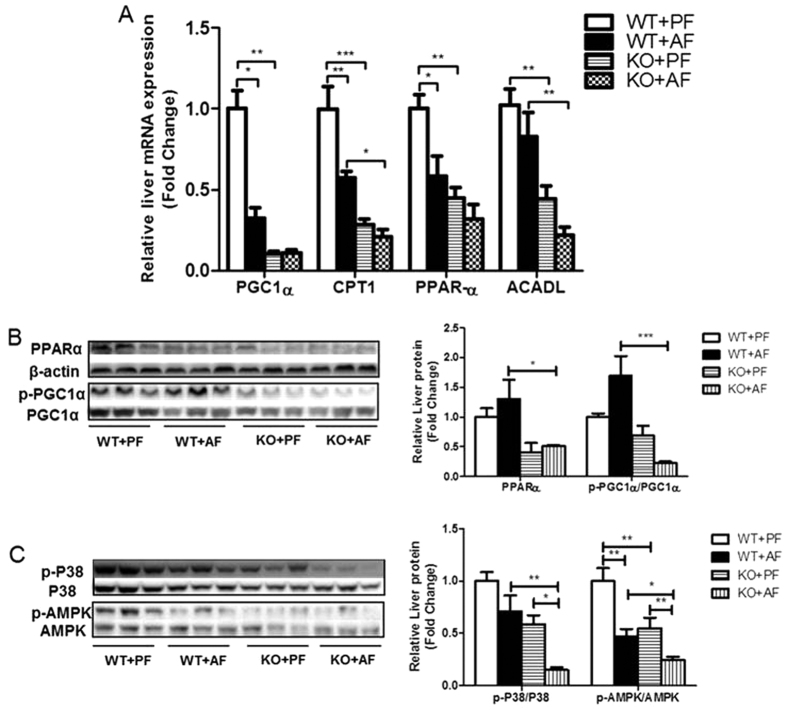
FGF21 deficiency inhibits alcohol-mediated fatty acid β-oxidation. Mice were treated as described in Material and Methods. (**A**) Relative liver mRNA levels of PGC1α, CPT1, PPARα, and ACADL. (**B**) Liver protein levels of PPARα, p-PGC1α and total PGC1α (left panel), and the ratios of PPARα/β-actin and p-PGC1α/PGC1α (right panel). (**C**) Liver protein levels of p-p38, p-AMPK and total p38, AMPK.

Alcohol exposure stimulates hepatic fatty acid accumulation via inhibition of fat clearance mediated by fatty acid β-oxidation. Therefore, we investigated whether FGF21 is critically involved in fatty acid clearance. Alcohol decreased the transcript levels of genes involved in fatty acid β-oxidation, including PGC1α, PPARα, and carnitine palmitoyltransferase I (CPT1), and these decreases were further exaggerated in the KO mice (Fig. 5A). The mRNA expression of long chain acyl-CoA dehydrogenase (ACADL), a mitochondrial enzyme that catalyzes most fatty acid β-oxidation, was not changed by alcohol in the WT, but significantly decreased in the KO mice (Fig. 5A).

PGC1α is a member of a family of transcription co-activators that play a central role in the regulation of cellular energy metabolism. In the liver, induction of PGC1α stimulates the PPARα-mediated transcription of genes involved in fatty acid oxidation. Alcohol exposure markedly decreased PPARα protein levels in the KO mice (Fig. 5B). Importantly, PGC1α phosphorylation was reduced in the KO mice (Fig. 5B), indicating a decrease in PGC1α activity in the AF KO mice.

PGC1α activation by phosphorylation is mediated by multiple mechanisms. Previous studies demonstrated that p38 MAPK activation directly phosphorylates PGC1α in the liver^13^. Alcohol exposure moderately decreased hepatic p38 phosphorylation in WT mice, but significantly in the KO mice (Fig. 5C). AMPK is a key metabolic master switch which phosphorylates target molecules involved in lipid metabolism in the liver^4^. As expected, alcohol exposure markedly decreased AMPK phosphorylation in WT mice. Importantly, the p-AMPK level was further down-regulated in FGF21 KO mice (Fig. 5C). These results strongly suggest that FGF21 plays a critical role in p38- and AMPK- mediated PGC1α-PPARα activation that regulates the genes involved in fatty acid β-oxidation in response to alcohol exposure.

### FGF21 KO increases chronic alcohol exposure-induced inflammation

**Figure 6:**
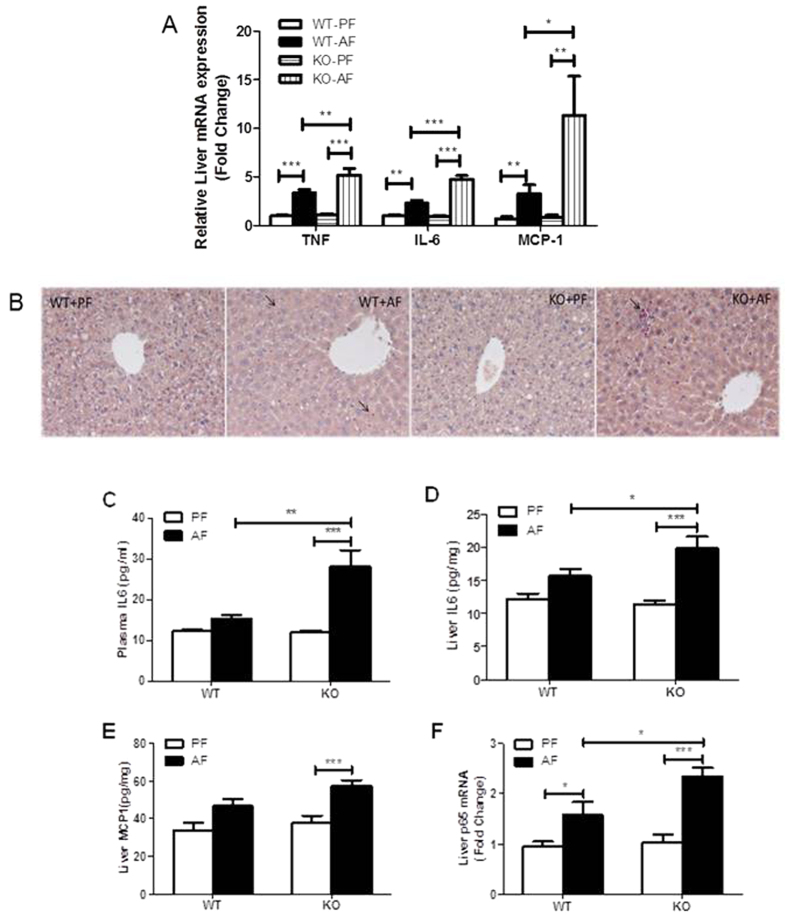
FGF21 KO increases chronic alcohol exposure-induced inflammation. Mice were treated as described in Material and Methods. (**A**) Relative liver mRNA levels of TNFα (left panel), IL6 (middle panel) and MCP1 (right panel). (**B**) Liver inflammation was assessed by CAE staining of liver sections. Arrows denote neutrophil infiltration. (**C**) Plasma IL6 protein concentrations. Liver IL6 (**D**) and MCP1 (**E**) protein levels, expressed in pg/mg total protein. (**F**) Relative liver mRNA levels of p65.

Inflammation is a hallmark of alcoholic liver disease. We have previously shown that alcohol exposure increased levels of circulating endotoxin, which activates Kupffer cells in the liver and increases hepatic inflammation^14^,^15^. The hepatic gene expression of pro-inflammatory cytokines, TNFα (tumor necrosis factor α), IL6 (interleukin 6) and chemokine MCP1 (monocyte chemoattractant protein 1) was significantly elevated in FGF21 KO mice exposed to alcohol compared with WT mice (Fig. 6A). The increase of plasma IL-6 protein (Fig. 6C) and hepatic levels of IL-6 and MCP1 protein was further enhanced in the KO mice (Figs 5E and 6D). The increase of cytokines and chemokines suggest an enhanced involvement of lymphocyte recruitment in the KO mice fed alcohol. In addition, chronic alcohol exposure also increased neutrophil infiltration in the liver of WT mice, and this increase was further exacerbated in FGF21 KO mice (Fig. 6B). Therefore, the observed effects of alcohol exposure on hepatic inflammation may be mediated, at least in part, by FGF21 signaling. In addition, alcohol exposure increased hepatic p65 gene expression, and this increase is further exacerbated in the KO mice (Fig. 6F). This finding indicates that increased hepatic inflammation by chronic alcohol may be mediated through FGF21 signaling.

### Recombinant FGF21 attenuates chronic alcohol-induced hepatic steatosis and injury

**Figure 7:**
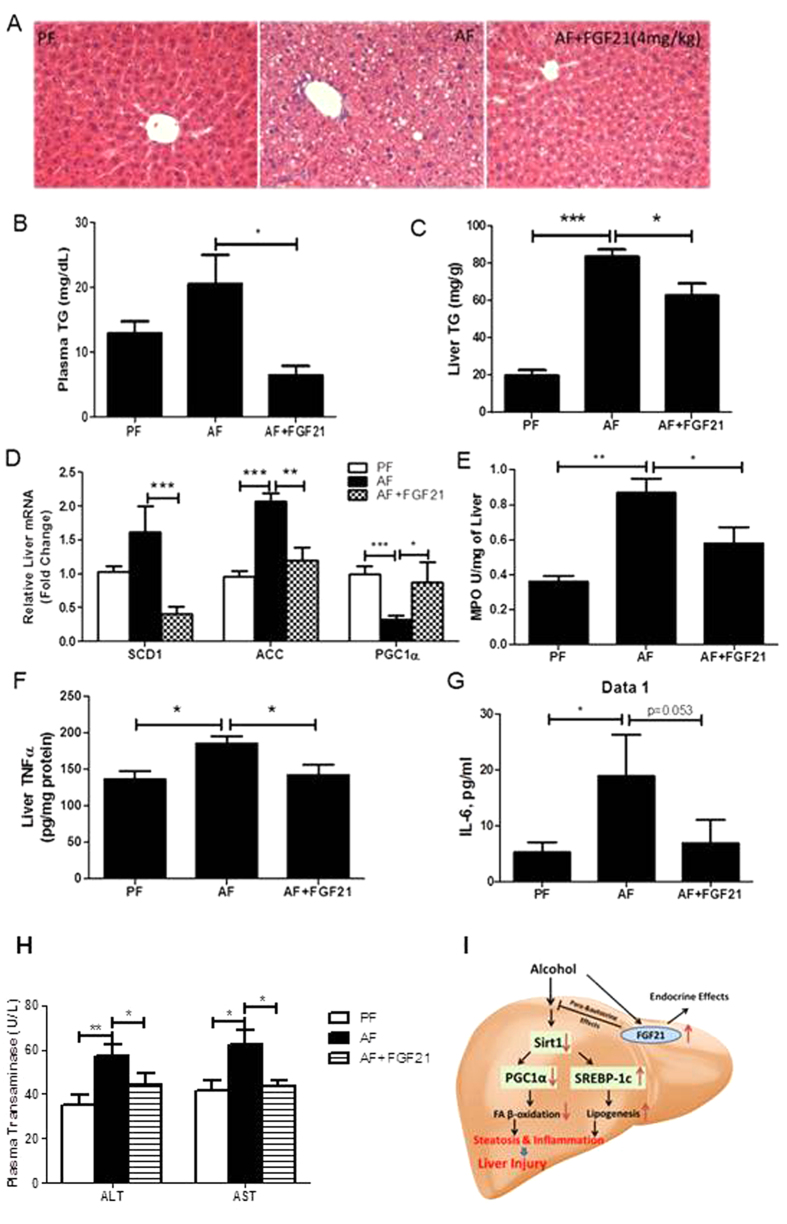
Recombinant FGF21 treatment attenuates chronic alcohol-induced hepatic steatosis and injury. WT mice were treated as described in Material and Methods. (**A**) H&E staining of liver sections. (**B**) Plasma TG levels. (**C**) Liver TG levels. (**D**) Relative liver mRNA levels of SCD1, ACC and PGC1α. (**E**) Liver MPO activity. (**F**) Liver TNFα protein levels. (**G**) Plasma IL-6 levels. (**H**) Plasma ALT and AST levels. (**I**) Schematic illustration of hypothesized mechanisms. Alcohol exposure increases circulating levels and hepatic expression of FGF21, which inhibit alcohol-induced down-regulation of Sirt1 leading to an increased fatty acid β-oxidation and a decreased lipogenesis mediated by PGC-1α and SREBP-1, respectively, and a reduced inflammation.

Based on above findings, we hypothesized that FGF21 may be beneficial in the treatment of ALD. To test this hypothesis, we injected rhFGF21 once a day at a dose of 4 mg/kg in the last 5 days of alcohol feeding. rhFGF21 treatment significantly attenuated chronic alcohol-induced hepatic fat accumulation (Fig. 7A). This effect was confirmed by the measurement of liver/body weight ratio (Supplementary Figure 5) and liver TG concentrations (Fig. 7C). Plasma TG concentrations were also significantly decreased by rhFGF21 treatment (Fig. 7B). rhFGF21 treatment resulted in an increase in the gene expression of PGC1α, and a decrease of SCD1 and ACC (Fig. 7D). To evaluate hepatic inflammation, we measured hepatic Myeloperoxidase (MPO) activity and cytokine expression. As expected, hepatic MPO activity (Fig. 7E), TNFα protein levels (Fig. 7F) and plasma IL-6 levels (Fig. 7G) were decreased by rhFGF21 treatment. Lastly, chronic alcohol-induced elevations in plasma levels of ALT and AST (aspartate aminotransferase) were markedly reduced by rhFGF21 treatment (Fig. 7H). These results suggest that FGF21 administration attenuates the chronic alcohol-induced liver steatosis and injury.

## Discussion

Previous studies have shown that FGF21 is a critical regulator for glucose and lipid metabolism in response to a variety of physiological conditions and pathological challenges. The present study provides evidence for the possible involvement of FGF21 signaling in the development of chronic alcohol-induced hepatic steatosis and injury. Several lines of evidence support this notion. First, we showed that circulating FGF21 levels are increased in patients with alcoholic steatohepatitis and that chronic alcohol exposure resulted in an up-regulation of FGF21 expression in mice. Second, FGF21 KO mice were sensitized to alcohol exposure with regard to hepatic lipogenesis and fatty acid β-oxidation leading to an increased hepatic fat accumulation. Third, FGF21 KO mice also exhibited an enhanced hepatic inflammation in response to alcohol. Fourth, rhFGF21 treatment reversed alcohol-induced hepatic steatosis and liver injury in mice.

Multiple studies suggest that a variety of cell types express FGF21 as an endocrine or paracrine/autocrine hormone with various functions. Serum FGF21 level is positively correlated with hepatic fat content and serum triglyceride concentration^16^,^17^,^18^. Inflammation has been shown to induce hepatic FGF21 expression^19^. Alcohol exposure causes hepatic fat accumulation and inflammation, which are clearly causative factors for FGF21 elevation in steatohepatitis patients and in mice. Although they have severe inflammation, cirrhotic patients have minimum hepatic fat, which may be responsible for the unchanged serum FGF21 level in cirrhotic patients. In addition, our results suggest that the liver is one of the major producing organs for circulating FGF21 induced by alcohol exposure. Liver insufficiency in cirrhotic patients apparently is a major factor for the irresponsibility in FGF21 production.

Hepatic FGF21 expression is under the control of PPARα in response to fasting^20^, while adipose expressed FGF21 functions locally, serving as an autocrine factor stimulating peroxisome proliferator-activated receptor γ (PPARγ) activity^21^. However, hepatic protein levels of PPARα are decreased by alcohol exposure, suggesting that PPARα is unlikely to be a major mediator in alcohol-induced hepatic FGF21 expression. Previous studies also identified that FGF21 expression is regulated, either positively or negatively by activating transcription factor 4 (ATF4), liver X receptor (LXR), carbohydrate-responsive element-binding protein (ChREBP) or farnesoid X receptor/retinoid X receptor-α (FXR/RXRα) under multiple pathological or physiological conditions^22^. In particular, application of the endoplasmic reticulum (ER) stressor, tunicamycin, induced hepatic FGF21 expression in mice and a marked elevation of serum FGF21 levels, which can be mimicked by overexpression of ATF4. Studies also indicate that FGF21 expression is induced by ATF4 and C/EBP homologous protein (CHOP)^23^, which are two important transcription factors involved in ER stress. Interestingly, previous studies demonstrated that alcohol exposure induces hepatic ER stress in humans^24^, experimental animal models^25^, and in cultured hepatocytes^26^. It is likely that the alcohol-induced FGF21 expression is regulated by ER stress response in the liver. In fact, the ATF4 DNA binding site has been identified in the FGF21 promoter^23^. Despite the up-regulation of FGF21 expression, treatment with recombinant FGF21 attenuates obesity- and diabetes-induced glucose and lipid dysregulation. Similar to insulin resistance, the enhanced expression of FGF21 has been attributed to a likely “FGF21 resistant state” in obese animals^27^. However, this FGF21 resistant state was not confirmed by other investigators^28^, leaving a debate on the nature of FGF21 regulation in the metabolic syndrome. Our results showed that that alcohol exposure and subsequent rhFGF21 treatment enhanced ERK phosphorylation in H4IIE cells (Supplementary Figure 6), suggesting that alcohol-exposed hepatocytes are not in a FGF21 resistant state. Therefore, exogenous FGF21 could potentially be used as a treatment for alcoholic fatty liver disease.

The enhanced FGF21 expression induced by alcohol is an adaptive response to stimulate cellular defenses against lipid dysregulation. Lack of this adaptive ability in FGF21 KO mice further exacerbated alcohol-induced liver steatosis. These compensatory effects of FGF21 have been demonstrated by others. Up-regulation of FGF21 expression has been shown to protect *ob/ob* mice from toxicity of sepsis^19^, and acetaminophen-induced liver injury^29^ in mice. A recent study demonstrated that loss of FGF21 induction in general control nonderepressible 2 (GNC2) knockout mice resulted in an exaggerated hepatic steatosis further supporting the notion that FGF21 is important in stimulating cellular defenses against lipid dysregulation^30^.

The protective effects of FGF21 in AFLD seem to be closely correlated with *de novo* lipogenesis and fatty acid catabolism. With increased hepatic steatosis, expression and the acetylation levels of hepatic SREBP1c were found to be up-regulated in alcohol exposed FGF21 KO mice leading to enhanced gene expression and activation involved in fatty acid *de novo* synthesis. On the other hand, hepatic PGC1α phosphorylation was severely decreased in FGF21 KO mice exposed to alcohol, leading to a reduction of the expression of genes encoding the molecules responsible for fatty acid β-oxidation. These findings suggest that the loss of FGF21 leads to the dysregulation of lipid metabolism in response to alcohol exposure. Indeed, FGF21 has been shown to regulate these anabolic and catabolic genes at the transcription level in obese and diabetic animals^31^. Phosphorylation of PGC1α increases its ability to interact with PPARα leading to the transcriptional activation^32^. Both AMPK and p38 MAPK phosphorylate PGC1α^13^. We found that phosphor AMPK and p38 levels were severely reduced in FGF21 KO mice exposed to alcohol, indicating a link between FGF21 and AMPK- and p38-medaited PGC1α activation in alcoholic fatty liver. In addition, high SCD-1 contributes to the suppression of AMPK activity^33^. Therefore, our results can be interpreted to indicate that AMPK and p38 activation mediates the effect of FGF21 on PGC1α activation leading to alteration in fatty acid catabolism in alcoholic fatty liver.

Interestingly, FGF21 ablation markedly decreased the activation of Sirt1 which is a known deacetylase targeting a variety of molecules including SREBP1c and PGC1α. Therefore, it is likely that FGF21 mediates the suppression of hepatic SREBP1c^34^,^35^ and activation of PGC1α^36^ through Sirt1 activation in response to alcohol exposure in mice.

A surprising finding was the role of FGF21 in the suppression of alcohol-induced hepatic inflammation. Genetic ablation of FGF21 significantly increased the expression of proinflammatory cytokines in mice exposed to alcohol, suggesting that FGF21 is potentially anti-inflammatory. Our results showed that the anti-inflammatory property of FGF21 was likely mediated by suppression of NFkB activity. Correlating with this finding, a previous study demonstrated that inflammation increases FGF21 expression, which is likely a feedback response to suppress inflammation^19^. Additional studies are needed to evaluate the potential action of FGF21 in the suppression of the activity of NFκB and attenuation of pro-inflammatory cytokines in response to endotoxin and alcohol exposure in Kupffer cells.

The role of FGF21 in ALD appears to be alcohol exposure pattern dependent. Acute alcoholic fatty liver was caused mostly by taking up the mobilized FFAs from adipose tissue^37^; and chronic alcoholic fatty liver involves *in situ* lipogenesis in addition to the increased mobilization of FFAs from adipose tissue^38^. We have demonstrated that chronic-binge alcohol exposure upregulates FGF21 expression which stimulates catecholamine release and enhances adipose tissue lipolysis, leading to increased fat accumulation in the liver^39^. However, in the chronic alcohol exposure model, the enhanced adipose lipolytic effect of FGF21 was overridden by the attenuated *in situ* hepatic lipogeneses and enhanced fatty acid β-oxidation. Further studies dissociating the role of FGF21 in adipose and liver using a tissue specific knockout strategy in ALD are needed.

A limitation in current study is that the Lieber DeCarli chronic alcohol feeding mouse model induces steatosis with only mild liver injury and inflammation. The protective effects, in particular, the anti-inflammatory effect, of FGF21 need further investigation in more severe rodent models of ALD and in humans to precisely define the potential role of FGF21 in advanced ALD. Unfortunately, there are no experimental models that recapitulate the full progression of ALD in humans.

Regardless this limitation, our findings are relevant to human ALD. Circulating levels of FGF21 are elevated in patients with alcoholic steatohepatitis but not cirrhosis, implying interplay between hepatic fat content and FGF21 expression. Recently, an FGF21 analog, LY2405319, has been developed and used in a randomized, placebo-controlled, double-blind trial in patients with obesity and type 2 diabetes. Patients receiving LY2405319 displayed a significant improvement in dyslipidemia^40^, indicating a role of FGF21 in lipid homeostasis in humans. Therefore, FGF21 could potentially be used to treat patients with ALD. Of note, during the submission period, Zhu *et al.* published a paper showing that FGF21 treatment ameliorates ALD in mice^41^.

In summary, we demonstrated that loss of FGF21 leads to worsened steatohepatitis in mice chronically exposed to alcohol. Our findings provide novel insights into the functional role of FGF21 in ALD. The effects of FGF21 on AFLD are attributed to multiple factors including the involvement of FGF21 in p38- and AMPK-mediated PGC1α activation in fatty acid catabolism and acetylation of SREBP1c via Sirt1 in fatty acid *de novo* synthesis and inflammation (Fig. 7I). The present study suggests that FGF21 treatment reversed the development of experimental ALD and thus prevented the progression of fatty liver to advanced liver disease. Our findings suggest that developing a strategy targeting FGF21 to treat alcoholic steatoheptitis may be warranted.

## Methods

### Human studies

Written informed consent was obtained from all participants. All experiments were conducted in accordance with the guidelines of human research and were approved by Clinical Research Ethics Committees of the University of Louisville and Robley Rex VA Medical Center, Louisville, KY, USA. Samples from patients with alcoholic steatohepatitis (ASH) were selected from a large specimen bank of ASH patients. Patients with severe ASH included in this report all had a liver biopsy during their hospitalization, and a subset of 24 subjects not having underlying cirrhosis was included. All ASH patients had clinical and biochemical evidence of alcoholic hepatitis and further baseline demographic information is provided in Supplementary Table 1. ASH patients were consuming ~46% of total calories (1226 Kcal) as alcohol prior to hospitalization, and they had a mean alcohol abuse history of 24 years. Exclusion criteria included: other liver diseases, including viral and metabolic; underlying cancer, and/or active infection. All subjects were active drinking within 1 month of hospitalization.

Twenty patients with alcoholic cirrhosis (AC) had testing supporting a diagnosis of cirrhosis including low platelet count and history or present findings of ascites or esophageal varices, hepatomegaly, history of chronic alcohol intake (>40 g/day for >5 years), as well as exclusion of other causes of cirrhosis, including viral and metabolic. In patients in whom the diagnosis remained uncertain, liver biopsy was performed for histologic confirmation. All subjects were Child-Turcotte-Pugh A or B. Subjects were not actively drinking at the time of study inclusion.

Healthy volunteers were age-, sex-, and BMI-matched to subjects with liver diseases. None of the volunteers had a history of active liver disease.

### Animal Studies

Male C57BL/6J mice (wild type, WT) were obtained from Jackson Laboratory (Bar Harbor, Maine). FGF21 KO mice were provided by Dr. Steve Kliewer^42^. All mice were bred in the University of Louisville animal vivarium. The KO mice were back-crossed at least 6 generations onto the C57BL/6 background. Male mice of WT and KO were divided into two groups at 8–10 weeks of age: Lieber DeCarli alcohol diet (alcohol-fed, AF) and isocaloric maltose-dextrin diet (pair-fed, PF) (Bio-Sev, Frenchtown, NJ), as described previous^14^. For the induction of ALD, mice were fed Lieber DeCarli diet with gradually increased alcohol concentration in the first 6 days to reach 5% (w/v) alcohol and were continually on the diet for 28 days. The diet composition was as described as previously^43^. One additional group of alcohol-exposed mice was treated with 4 mg/kg recombinant human FGF21 (rhFGF21)^44^ via intraperitoneal injection in the last 5 days. At the end of the experiment, the mice were anesthetized with avertin (2, 2, 2-tribromoethanol). Plasma and tissue samples were collected for assays. All mice were treated according to the protocols reviewed and approved by the Institutional Animal Care and Use Committee of the University of Louisville.

### Blood biochemical assays

Mouse blood samples were centrifuged at 1500 *g* for 30 min at 4 °C to obtain plasma. Plasma variables were measured using commercial kits closely following the manufacturer’s instructions. Alanine aminotransferase (ALT) and aspartate aminotransferase (AST) levels were measured using ALT and AST Assay Kits (Thermo Fisher Scientific Inc., Middletown, VA). Free fatty acids, glycerol, cholesterol and triglyceride levels were quantified using commercial kits (Wako Chemicals, Richmond, VA). IL6 and MCP1 concentrations were measured using commercial kits (Life technologies, Gaithersburg, MD, USA). Mouse blood alcohol concentrations were measured using a commercial kit (Abcam, Cambridgeshire, UK).

FGF21 concentrations in the plasma of mice and in serum of humans were measured using ELISA kits from Biovendor, Modrice, Czech Republic and R&D, Minneapolis, MN, respectively.

### Liver triglyceride assay

For the liver triglyceride assay, 70–100 mg of liver tissue was homogenized in 1 ml of 50 mM NaCl. Homogenate (500 μl) was mixed with 4 ml of the extraction reagent (methanol: chloroform = 1:2) and incubated overnight at 4 °C before being centrifuged at 1,800 *g* for 20 min at room temperature. The lower chloroform phase was carefully collected and dried using a Speed Vac, and the pellets were used for triglyceride assay using the Triglyceride Kit (Thermo Fisher Scientific Inc.).

### Determination of hepatic cytokine and chemokine concentration

Fifty to seventy mg liver tissue was homogenized in RIPA buffer (50 mM Tris·HCl, pH 7.4, 150 mM NaCl, 2 mM EDTA, 4 mM Na3VO4, 40 mM NaF, 1% Triton X-100, 1 mM phenylmethylsulfonyl fluoride, 1% protease inhibitor cocktail)^14^. TNFα, IL6 and MCP1 protein levels were measured using their respective ELISA kits (BD, Sparks, MD, USA) according to the manufacturer’s instructions. The values were expressed in pg/mg total protein.

### Liver histology and fat analyses

The liver sections were fixed in formalin and embedded in paraffin. The sliced liver sections were then stained with H&E as described previously^14^. For hepatic fat visualization, frozen liver sections were processed for staining with Oil red O and then studied by light microscopy^43^.

### Liver neutrophil accumulation

Formalin-fixed paraffin-embedded liver sections were deparaffinized and rehydrated. Neutrophils were stained using a naphthol AS-D chloroacetate (Specific Esterase) (CAE) staining kit (Sigma-Aldrich, St. Louis, MO) according to the manufacturer’s directions^45^.

### TUNEL assay

Formalin-fixed paraffin liver sections were sectioned at 5 μm. The sections were stained for TUNEL with the ApopTag Peroxidase *in situ* Apoptosis Detection Kit (Chemicon, CA, USA). In brief, the slides were deparaffinized and rehydrated, then treated with proteinase K (20 μg/ml) for 15 min at room temperature. Slides were treated with 3% hydrogen peroxide for 5 min to quench endogenous peroxidases, and then incubated with terminal deoxynucleotidyl transferase (TdT) and anti-digoxigenin-peroxidase at 37 °C for 1 h or 30 min respectively. Diaminobenzidine (DAB) was then applied. Hematoxylin was used as counterstaining. Under the microscope, apoptotic cells exhibited a brown nuclear stain as the TUNEL positive and were counted manually.

### Quantitative real time RT-PCR

The mRNA levels were assessed by real-time RT-PCR. In brief, total RNA was isolated with Trizol according to manufacturer’s protocol (Invitrogen, Carlsbad, CA) and reverse-transcribed using GenAmp RNA PCR kit (Applied Biosystems, Foster City, CA). The cDNA was amplified in 96-well reaction plates with a SYBR green PCR Master Mix (Applied Biosystems) on an ABI 7500 real-time PCR thermocycler. The sequences of forward and reverse primers are listed in Supplementary Table 2. The relative quantities of target transcripts were calculated from duplicate samples after normalization by a housekeeping gene, β-actin. Dissociation curve analysis was performed after PCR amplification to confirm the specificity of the primers. Relative mRNA expression was calculated using the ^ΔΔ^Ct method.

### Immunoprecipitation and Western blot analysis

For immunoprecipitation, 1 mg of tissue lysate was incubated with 2 μg of antibody (anti-SREBP1c) at 4 °C overnight. After the addition of 40 μl PureProteome™ Protein A/G Mix Magnetic Beads (Millipore), incubation was continued for an additional 2 h at 4 °C. The beads were then collected by magnet and washed three times with washing buffer (PBS containing 0.1% Tween 20). 2x SDS sample buffer was added to the beads and incubated at 95 °C for 10 min, and Western blot was then performed. Acetylated SREBP1c was detected by an anti-acetylated lysine antibody (Cell Signaling, Danvers, MA, USA).

Western blot was performed as described previously^14^ to detect precursor and mature forms of SREBP1c (Santa Cruz Biotechnologies, Santa Cruz, CA), pAMPK, AMPK, p-p38, p38, SCD1, Sirt1, acetylated-lysine (Cell Signaling Technologies), FGF21 (Abcam, San Francisco, CA), PPARα, PGC1α, β-actin (Santa Cruz Biotechnology), p-PGC1α (R&D, Minneapolis, MN). Blots were scanned using a Bio-Rad Imaging System (Image Lab™ Upgrade for ChemiDoc™ XRS + System #170-8299). All specific bands were quantified with the Automated Digitizing System (Image Lab 4.1). Results are representative of three independent experiments.

### Isolation and culture of primary hepatocytes

Hepatocytes were isolated from the WT and FGF21 KO mice by *in situ* digestion of the liver with perfusion of collagenase type IV. Briefly, total liver tissues were perfused with EGTA solution (10 mM HEPES [pH 7.4], 5 mM glucose, 138 mM NaCl, 5.4 mM KCl, 28.3 mM NaHCO_3_, 0.12 mM Na_2_HPO_4_, 0.56 mM NaH_2_PO_4_ and 0.5 mM EGTA) into the inferior vena cava. After perfusion, the liver tissues were dissociated into hepatocytes using collagenase solution (10 mM HEPES [pH 7.4], 138 mM NaCl, 5.4 mM KCl, 28.3 mM NaHCO_3_, 0.12 mM Na_2_HPO_4_, 0.56 mM NaH_2_PO_4_ supplemented with 0.0857 U/ml type IV collagenase (Roche Diagnostics, Indianapolis, IN) and 3.8 mM CaCl_2_). Subsequently, the isolated hepatocytes were washed with serum-free Waymouths medium (Gibco BRL, Life Technologies, Inc., Grand Island, NY) and suspended in Waymouths medium supplemented with 10% (w/v) fetal bovine serum (FBS) (Gibco BRL, Life Technologies, Inc.), Antibiotic-Antimycotic (Gibco 100 units/mL of penicillin, 100 μg/mL of streptomycin, 0.25 μg/mL of Fungizone) and ITS supplement (VWR). Cell viability was assessed by the trypan blue exclusion test. Isolated hepatocytes were seeded at a density of 3.5 × 10^5^ cells/dish in 35-mm tissue culture dishes and maintained at 37 °C in 5% CO_2_. After cell attachment (approximately 4 hours), the culture media were replaced with fresh media for treatment. All cell culture experiments were carried out with the guidelines of biosafety and approved by the Biosafety Committee of the University of Louisville.

### Cell culture and treatment

Mouse AML-12 hepatocytes were provided by Dr. Min You at Northeast Ohio Medical College and were cultured in DMEM/F12 medium (ATCC) supplemented with 10% FBS, 100 μg/ml streptomysin, 100 Unit/mL penicillin, 0.1 μM dexamethasone, and insulin-transferrin-selenium (ITS; Life technologies). Cells were treated with rhFGF21 (1 μg/ml and 3 μg/ml) for 24 hours. Fixed cells were then incubated with anti-Sirt1 antibody (1:100, Cell Signaling) overnight at 4 °C. Alexa Fluor 488 goat anti-rabbit IgG(H + L) (1:500, Life Technologies) was used to visualizing Sirt1 expression. DAPI was used to detect nuclei. Images were obtained with EVOS^®^ FL Cell Imaging System (Life Technologies).

H4IIE cells were purchased from ATCC and maintained in 10% FBS in Eagle’s Minimum Essential Medium (ATCC) containing 100 Unit/mL penicillin and 10 μg/mL streptomycin at 37 °C under a 5% CO_2_ atmosphere. H4IIE cells were exposed to alcohol (100 mM) for 72 hours, and then incubated with rhFGF21 (1 μg/ml) for 5, 10, 20 or 60 minutes, respectively. ERK phosphorylation was detected by Western blot analysis.

### Statistical Analysis

Data are expressed as means ± SEM. Two-way ANOVA with Bonferroni post-test, or One-way ANOVA with Tukey post-test, or two-tailed unpaired Student’s t-test were used for the determination of statistical significance of the data where they were appropriate. All statistical analyses were performed with GraphPad Prism software Version 5 (GraphPad Software, Inc., San Diego, CA). Differences between groups were considered significant at *p < 0.05, **p < 0.01, ***p < 0.001. For animal study, n = 5–12 per group. For cell culture study, the experiments were performed in duplicate and repeated 3 times.

## Additional Information

**How to cite this article**: Liu, Y. *et al.* Fibroblast growth factor 21 deficiency exacerbates chronic alcohol-induced hepatic steatosis and injury. *Sci. Rep.* **6**, 31026; doi: 10.1038/srep31026 (2016).

## Supplementary Material

Supplementary Information
