# Supplementary material for: Fibroblast growth factor 21 deficiency exacerbates chronic alcohol-induced hepatic steatosis and injury
Source: Sci Rep. 2016 Aug 8;6:31026. doi: 10.1038/srep31026 (PMC4976373; doi:10.1038/srep31026)
Supplement: Supplementary Information [file srep31026-s1.pdf]

Supplementary Information for

Fibroblast growth factor 21 deficiency exacerbates chronic alcohol-induced hepatic steatosis and injury

Yanlong Liu[1,2,*], Cuiqing Zhao[1,2,3*] Jian Xiao[1*], Liming Liu[2], Min Zhang[2], Cuiling Wang[4], Guicheng Wu[2.5], Ming-Hua Zheng[1], Lan-Man Xu[1], Yong-Ping Chen[1], Moosa Mohammadi[6], , Shao-Yu Chen[2], Matthew Cave[2], Craig McClain[2,7], Xiaokun Li[1,3], Wenke Feng[1,2]

[1]School of Pharmacy and First Affiliate Hospital, Wenzhou Medical University, Wenzhou, Zhejiang, China, 325027, [2]Alcohol Research Center, University of Louisville School of Medicine, Louisville, KY, USA, 40202, [3]Institute of Life Sciences, Wenzhou University, Wenzhou, Zhejiang, China, 325035, [4]School of Life Sciences, Northwest University, Xi'an, Shaanxi, China, 710069 [5]Three George Central Hospital, Chongqing, China, 404000, [6]Department of Biochemistry and Molecular Pharmacology, New York University School of Medicine, New York, NY, USA, 10016, [7]Robley Rex VA Medical Center, Louisville, KY, USA, 40206

Supplementary Table 1. Demographic data and biochemical characteristics of human subjects

Group	Age	Gender (M/F)	BMI (Kg/m^2)	AST (U/L)	ALT (U/L)	Bilirubin	Albumin	MELD	Liver Biopsy (Y/N)
Control	57 ±7.4	22/4	25.2± 2.1	22.09± 1.116	13.91± 0.899	-----	-----	-----	0/26
ASH (n=24)	56.2 ±4.6	22/2	24.3± 2.3	139± 14	71± 17	18.1±2.2	2.9±1	26.3± 2.5	24/0

AC (n=20)	54.3 ±10	17/3	25.1± 22	52.05± 9.401	18.52± 1.878	1.2±0.2	3.5±0.3	8.9± 0.9	7/13

AC: Alcoholic Cirrhotics, ASH: Alcoholic Steatohepatitis, ALT, Alanine aminotransferase activity; AST, Aspartate aminotransferase activity; BMI: Body mass index, F: Female, M: Male, MELD: Model for End Stage Liver Disease.

Supplementary Table 2. Basic characteristics of WT and FGF21 KO mice exposed to chronic alcohol treatment

Characteristic	WT+PF	WT+AF	KO+PF	KO+AF
Body weight (g)	24.2±0.7	23.5±0.5	24.6±1.0	23.4±1.3
Fat weight (g)	0.62±0.05	0.35±0.04[a]	0.58±0.08	0.57±0.07[c]
Liver weight (g)	0.8±0.02	1.1±0.04[a]	1.0±0.14[b]	1.2±0.07[c,d]
Fat/Body weight (%)	2.4±0.19	1.2±0.13[a]	2.1±0.29	1.9±0.21[c]
Liver/Body weight (%)	3.5±0.11	4.6±0.1[a]	4.1±0.15[b]	5.0±0.13[c,d]
Food Intake (g/d per mouse)	10.5±0.85	10.7±0.98	10.2±0.56	10.6±0.78
Plasma EtOH (g/L)	ND	0.96±0.09	ND	0.94±0.16
Plasma TG (mg/L)	13.0±1.8	20.0±1.4	13.1±2.3	30.1±7.6[d]
Plasma FFA (mEq/L)	0.51±0.06	0.79±0.06[a]	0.37±0.02	0.72±0.09[d]
Plasma Glycerol (mg/L)	11.3±1.5	17.7±1.2[a]	13.7±1.2	15.2±1.8
Plasma Cholesterol (mg/ml)	229±17.9	257±9.33	277±23.4	239±37.8
Plasma Insulin (ng/ml)	0.62±0.08	0.58±0.09	0.62±0.10	0.61±0.04
Plasma Adiponectin (µg/ml)	13.1±0.56	11.8±0.47	12.2±0.19	11.8±0.16
Liver FFA (mEq/g liver)	0.46±0.07	0.73±0.05	0.46±0.06	0.97±0.22
Liver Cholesterol (mmol/g liver)	2.8±0.35	4.1±0.16[a]	2.8±0.09	3.9±0.19[d]

PA: Pair-Fed, AF: Alcohol-Fed, WT: Wild Type, KO: FGF21 knockout. Values are means± SEM. Significant difference: a: WT+PF vs. WT+AF, b: WT+PF vs. KO+PF, c: WT+AF vs. KO+AF, d: KO+PF vs. KO+AF (p<0.05)

Supplementary Table 3: Primer Sequences for Real-Time Quantitative RT-PCR analysis

Gene	Source	Sequences (Forward/Reverse 5'-3')	
ACC	Mouse	GGGACTTCATGAATTTGCTGATTCTCAGTT	GTCATTACCATCTTCATTACCTCAATCTC
ACADL	Mouse	TCTTTCCTCGGAGCATGACA	GACCTCTCTACTCACTTCTCCAG
ApoB	Mouse	TCCTGCTTCTGTTCTTGGACACCA	ACGTACTTCCGGAGGTGCTTGAAT
β-actin	Mouse	GGCTGTATTCCCCTCCATCG	CCAGTTGGTAACAATGCCATGT
β-Klotho	Mouse	GATGAAGAATTTCCTAAACCAGGTT	AACCAAACACGCGGATTTC
CD36	Mouse	GAACCACTGCTTTCAAAAACTGG	TGCTGTTCTTTGCCACGTCA
CPT1	Mouse	CCAGGCTACAGTGGGACA	GAACTTGCCCATGTCCTTGT
FAS	Mouse	TGGGTTCTAGCCAGCAGAGT	ACCACCAGAGACCGTTATGC
FATP2	Mouse	TCCTCCAAGATGTGCGGTACT	TAGGTGAGCGTCTCGTCTCG
FATP5	Mouse	CATCGCTGGCTGCATATAGATG	CCACAAAGGTCTCTGGAGGAT
FGF21	Mouse	CCTCTAGGTTTCTTTGCCAACAG	AAGCTGCAGGCCTCAGGAT
FGFR4	Mouse	CGCCAGCCTGTCACTATACAAA	CCAGAGGACCTCGACTCCAA
IL6	Mouse	ATGAAGTTCCTCTCTGCAAGAGAC	CACTAGGTTTGCCGAGTAGATCTC
MCP1	Mouse	GGCTCAGCCAGATGCAGT	GAGCTTGGTGACAAAAACTACAG
MTTP	Mouse	AAGGCCAATATGGACATCCAGGGT	TGGTTATTACCACAGCCACCCGAT
NFKB(P65)	Mouse	CTTGGCAACAGCACAGACC	GAGAAGTCCATGTCCGCAAT
PGC1α	Mouse	AGACAAATGTGCTTCCAAAAAGAA	GAAGAGATAAAGTTGGTTTGGC
PPARα	Mouse	AGAGCCCCATCTGTCCTCTC	ACTGGTAGTCTGCAAAACCAAA
SREBP1c	Mouse	GCGGAGCCATGGATTGCA	CTCTTCCTTGATACCAGGCCC
SCD1	Mouse	CCGGAGACCCTTAGATCGA	TAGCCTGATTTCTGCAAACC
Sirt1	Mouse	GTAAGCGGCTTGAGGG	TTCGGGCCTCTCCGTA
TNFα	Mouse	CACCACCATCAAGGACTCAA	AGGCAACCTGACCACTCTCC

Supplementary Figure 1

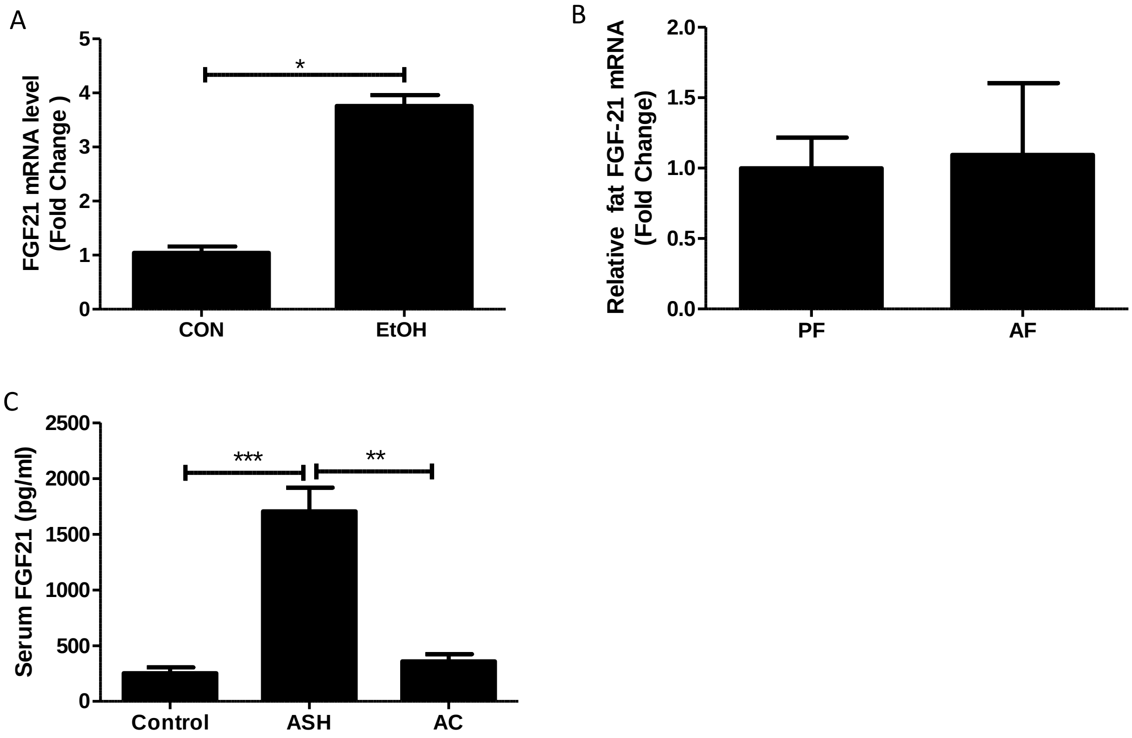

Supplementary Figure 1. Alcohol exposure increases FGF21 expression. (A) FGF21 mRNA levels of primary hepatocytes isolated from mice treated with alcohol at 6 g/kg body weight or isocaloric maltose dextrin by gavage. 6 hours after binge-alcohol treatment, the primary hepatocytes were isolated. (B) Mice were fed as described in Material and Methods. Relative mRNA levels of FGF21 in the epididymal fat tissue were measured by qRT-PCR. Results are expressed as mean ± SEM (n=5-12 mice). . (C) Serum levels of FGF21 in alcoholic steatohepatitis (ASH) and stable abstinent alcoholic cirrhosis (AC) patients. Patient demographic data and biochemical characteristics are listed in the Supplementary Table 1.

Supplementary Figure 2

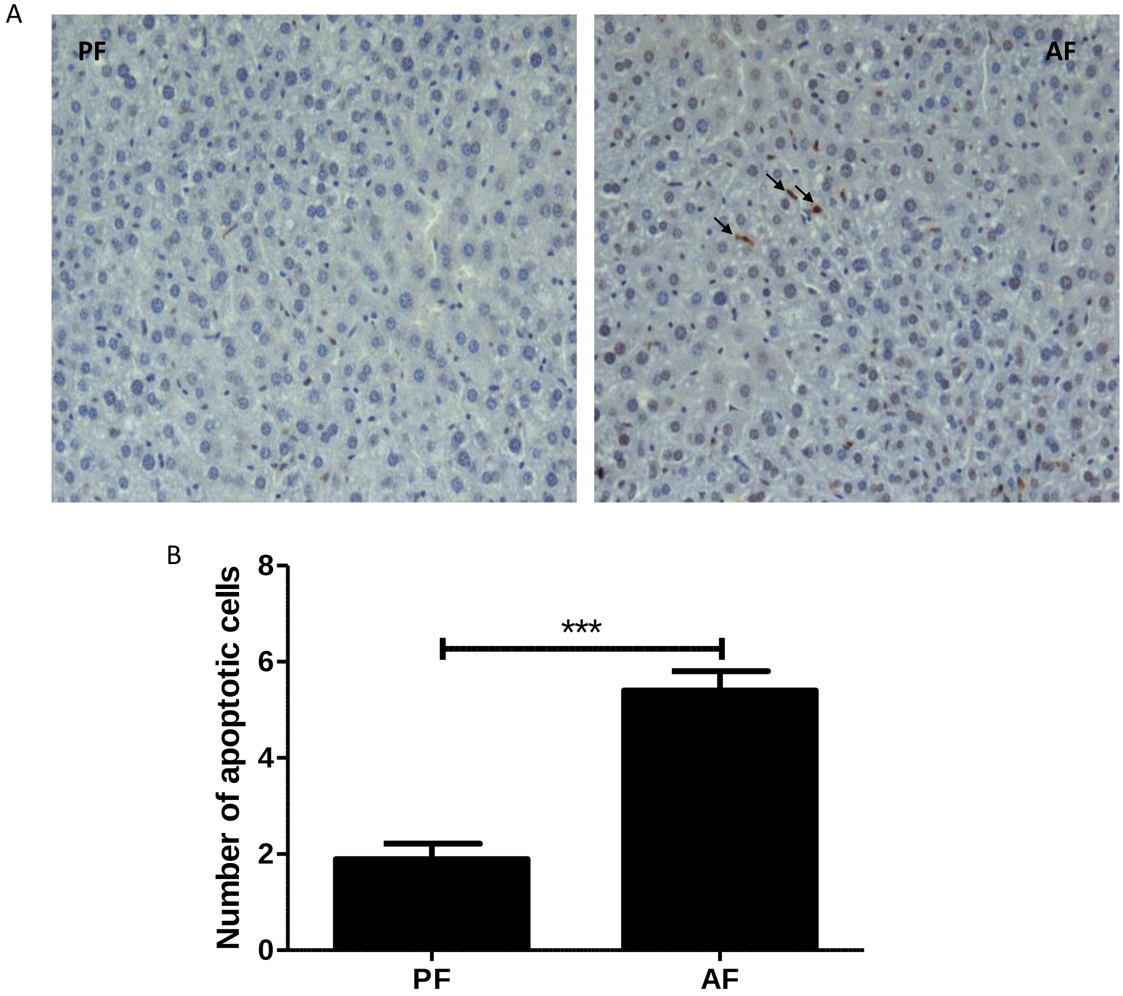

Supplementary Figure 2. Alcohol exposure induces liver apoptosis. Mice were fed as described in Material and Methods. (A) Representative images of terminal deoxynucleotidyl transferase dUTP nick end labeling (TUNEL) staining of liver sections. Arrows denote positive staining. (B) The number of apoptotic cells was determined by counting the number of TUNEL-positive cells in at least 10 randomly selected high-power fields (magnification, ×200).

Supplementary Figure 3

A

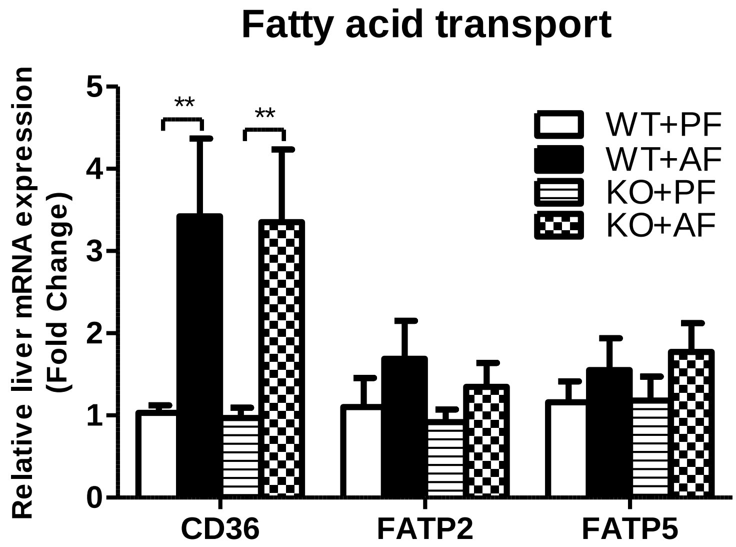

B

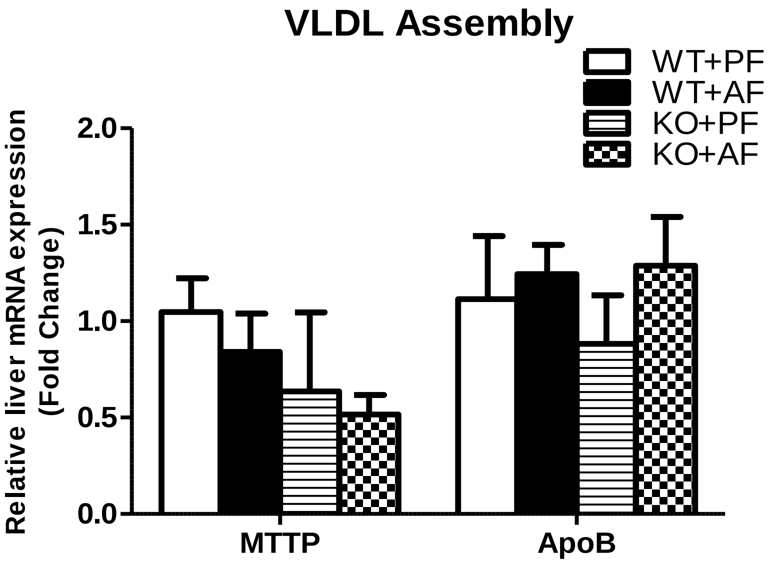

Supplementary Figure 3. FGF21 ablation does not alter the expression of genes responsible for fatty acid transport and VLDL assembly. Mice were fed as described in Material and Methods. (A) Relative liver mRNA levels of CD36, FATP2 and FATP5. (B) Relative liver mRNA levels of MTTP andApoB . Results are expressed as mean ± SEM (n=5-12 mice).

Supplementary Figure 4

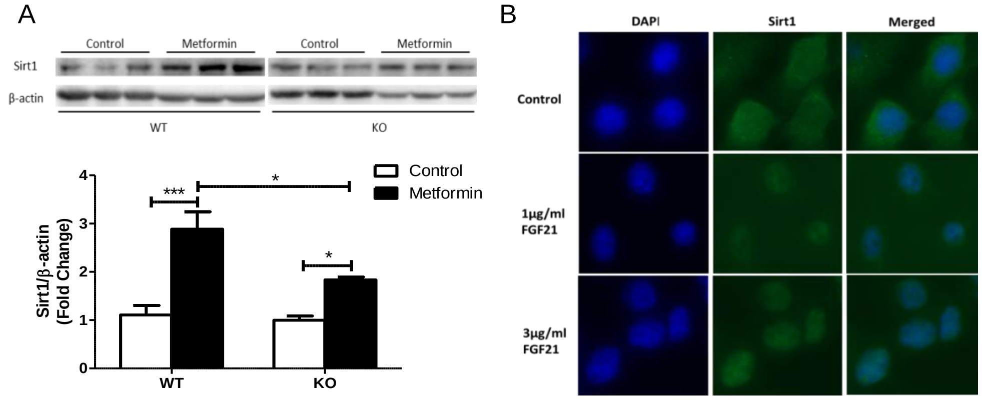

Supplementary Figure 4. SIRT1 expression and nuclear translocation in an FGF21-dependent manner in hepatocytes. (A) Sirt1 protein expression in primary hepatocytes exposed to 1 mM metformin for 8 hours. Western blotting was performed to measure Sirt1 protein levels. (B) AML-12 cells were treated with rhFGF21 for 24 hours. Cells were stained with anti-Sirt1 antibody. Nuclei were visualized by DAPI staining.

Supplementary Figure 5

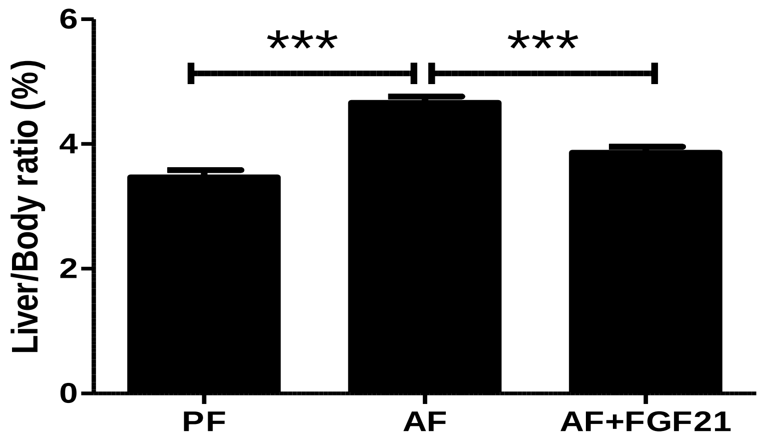

Supplementary Figure 5. Recombinant FGF21 treatment attenuates chronic alcohol-increased liver/body weight ratios.

Supplementary Figure 6

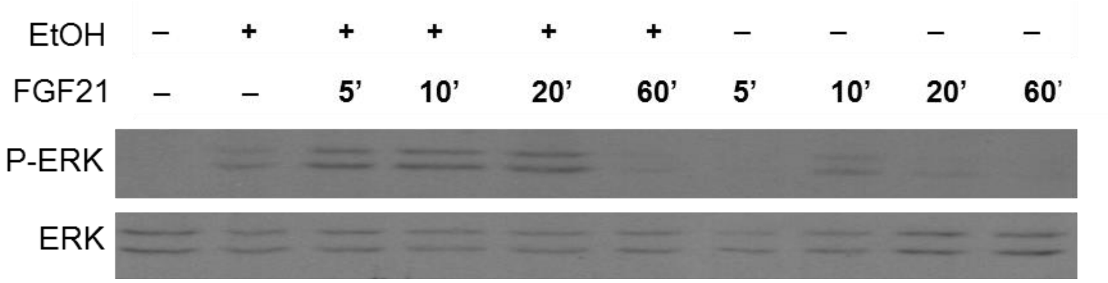

Supplementary Figure 6. Alcohol exposure enhances FGF21-induced ERK activation in hepatocytes. H4IIE cells were exposed to alcohol (100 mM) for 72 hours, and then incubated with rhFGF21 (1µg/ml) for the time intervals as indicated. ERK phosphorylation was accessed by Western blot analysis**.** The experiments were performed in duplicated and repeated at least 3 times.
